# Ventilation of a Dental Office

**Published:** 1902-06

**Authors:** Charles C. Linton

**Affiliations:** New York


### VENTILATION OF A DENTAL OFFICE.

BY DR. CHARLES C. LINTON, NEW YORK.

One of the most important things we have to contend with in our daily practice is the proper ventilation of our operating-rooms. As a rule, most office windows are opened only for a short time in the morning. This may be sufficient for each individual patient who remains in the room for an hour at a time, but it is not enough air for the operator who stays all day, breathing not only the same air over and over again, but more or less of the patients' breath, and this, together with the cramped position of the lungs, owing to the necessary stooping attitude the operator is obliged to frequently assume, makes it a very serious menace to his health, unless counteracted by a great amount of out-door exercise. In order to obtain the best results it is necessary to have a current of fresh air supplied continually.

Frequently there is but one window from which to ventilate, which makes the problem all the more difficult. I think I have solved this to a great extent by a very simple device. The method is not original with me, but is an old idea which I obtained from a magazine and put into practice. I had a carpenter fit a piece of board about three inches wide and the exact width of the window, and had it stained to match the wood-work, with a weatherstrip tacked on top. Then by raising the window and slipping into place, and closing the window on it, I have the ventilation between the two sashes above, through which I am constantly receiving a current of fresh air without a direct draught either over myself or my patient.

